# A Balancing Act: Learning from the Past to Build a Future-Focused Opioid Strategy

**DOI:** 10.1146/annurev-physiol-042022-015914

**Published:** 2023-11-29

**Authors:** Sarah Warren Gooding, Jennifer L. Whistler

**Affiliations:** 1 Center for Neuroscience, University of California, Davis, California, USA; 2 Department of Physiology and Membrane Biology, UC Davis School of Medicine, Davis, California, USA

**Keywords:** opioid, arrestin, G protein, signaling bias, receptor trafficking

## Abstract

The harmful side effects of opioid drugs such as respiratory depression, tolerance, dependence, and abuse potential have limited the therapeutic utility of opioids for their entire clinical history. However, no previous attempt to develop effective pain drugs that substantially ameliorate these effects has succeeded, and the current opioid epidemic affirms that they are a greater hindrance to the field of pain management than ever. Recent attempts at new opioid development have sought to reduce these side effects by minimizing engagement of the regulatory protein arrestin-3 at the mu-opioid receptor, but there is significant controversy around this approach. Here, we discuss the ongoing effort to develop safer opioids and its relevant historical context. We propose a new model that reconciles results previously assumed to be in direct conflict to explain how different signaling profiles at the mu-opioid receptor contribute to opioid tolerance and dependence. Our goal is for this framework to inform the search for a new generation of lower liability opioid analgesics.

## INTRODUCTION: A CENTURIES-LONG SEARCH FOR BETTER ANALGESICS

Opioid drugs are the standard of care for treating severe pain, making them some of the most widely used and clinically significant drugs in medicine. Many individuals who suffer from an opioid use disorder (OUD), colloquially known as addiction and broadly defined as the loss of control of drug seeking, were first exposed to opioid drugs in a clinical context. In this way, opioids differ from other drugs of abuse: Their use is often medically indicated and necessary. The molecular and neuronal mechanisms underlying the transition from opioid use to the opioid misuse/abuse that define an OUD remain poorly understood, but these mechanisms lie at the heart of the opioid epidemic. In the United States alone, there are more than 180 daily opioid overdose deaths, and the rate has been climbing since the 1990s and dramatically accelerated during the COVID-19 pandemic (1). The scale of this tragedy has promoted widespread interest in mitigating the side effects of opioid drugs so that their therapeutic benefits may be applied with a decreased risk to patients. Better drugs that relieve pain with minimal harmful side effects are the ultimate aim of analgesic drug development. To this end, much attention and research have been directed toward identifying ways to amplify the beneficial effects while reducing the side effects of opioid medications. Unfortunately, these efforts have met with little success. As we find ourselves in an age of unprecedented potential for drug development and screening, it is important not to forget what we have already learned from this field’s lengthy history and to incorporate those lessons into new approaches. Our endogenous opioid peptides already provide excellent pain relief without treatment-limiting side effects, a premise we find encouraging.

**Figure 1:**
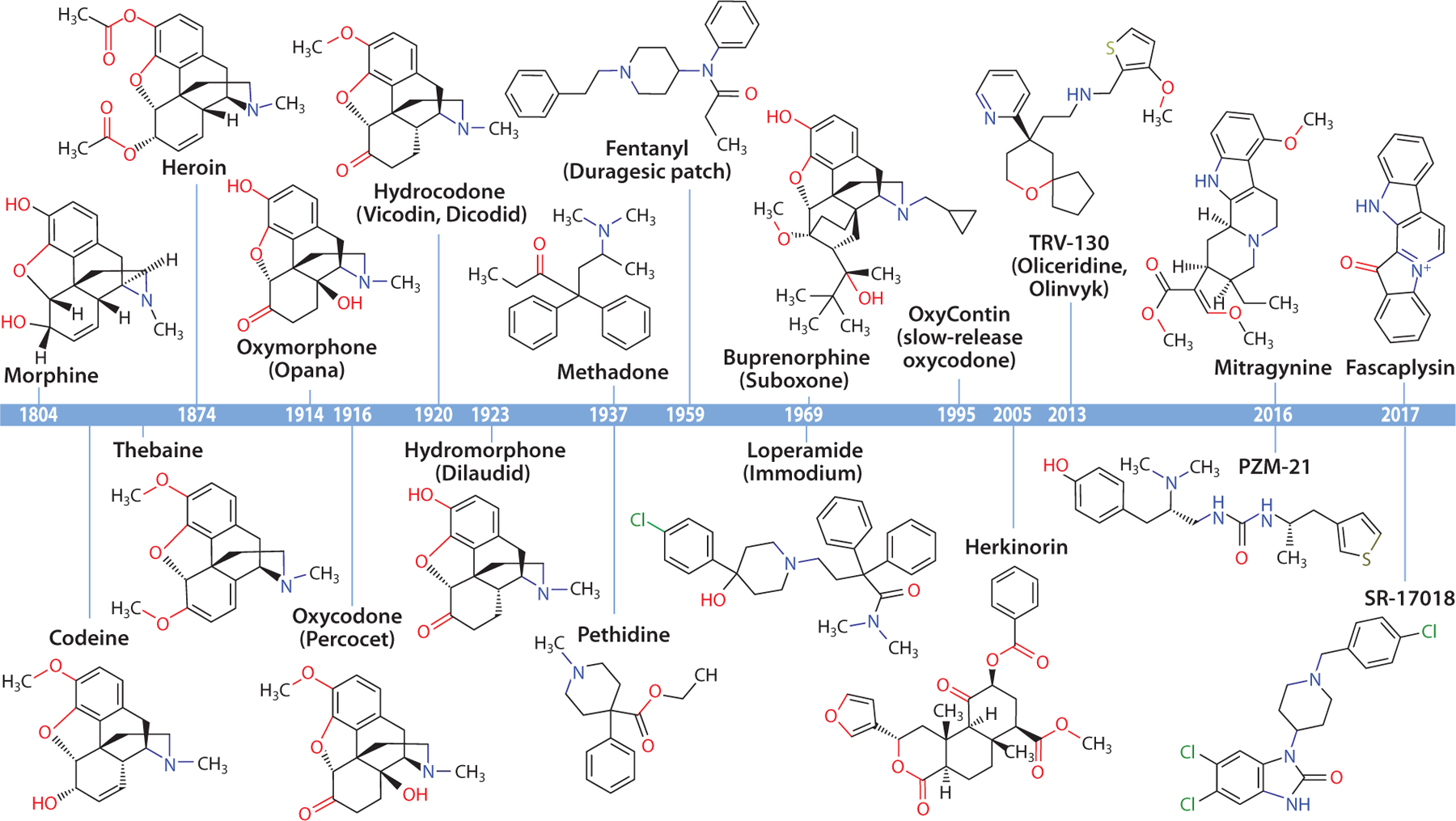
Historical timeline of opioid molecule discovery and synthesis.

Humans have used opioids for pain relief for thousands of years. The first known uses were of opium and tinctures of opium such as laudanum. It was in 1804 that the first active analgesic ingredient in opium, morphine, was isolated by Friedrich Sertürner (2). It was already recognized at that time that repeated use of opium resulted in addiction. Although Sertürner originally theorized that the need for smaller quantities of purified morphine compared to opium would reduce the addiction risk, this was not the case, and he documented his own addiction to morphine. The semisynthetic derivative of morphine, heroin (diacetylmorphine), was first synthesized in 1874 (3) and marketed by Bayer in 1898 as a less addictive opioid. This launched more than 100 years of false claims and false hopes for a painkiller with low abuse risk (Figure 1).

Additional early efforts to create less addictive opioids focused on various ways of derivatizing the natural opium extracts morphine and thebaine, creating several drugs we still use today. These include oxymorphone (1914, introduced as Opana in the United States in 1955), oxycodone (1916, first introduced in the United States in 1928 as part of Scophedal, then mixed with acetaminophen as Percocet, and more recently in a slow-release formula as OxyContin), hydrocodone (1920, approved in the United States in 1943 as Dicodid and mixed with acetaminophen as Vicodin), and hydromorphone (1923, marketed as Dilaudid in the United States from 1927) (4). Because these semisynthetic derivatives had abuse liability and still relied on precursors purified from a poppy, the next pharmaceutical quest was to identify fully synthetic opioids. The first synthetic opioid, pethidine/meperidine (sold as Demerol), with a structure unrelated to the morphine/thebaine opioids, was patented in 1937 and approved for use in 1943. However, its toxic metabolite counterindicates its use from long-term pain (4). Simultaneously, during a shortage of natural painkillers in World War 2, methadone was synthesized to reduce demand for opium and morphine (5). Methadone’s half-life is much longer than that of the natural product opioids, but its half-life is also highly variable in the human population (6). This variability has limited its use as a first-line analgesic, but it is still in widespread use for the treatment of OUD.

Two decades later, very high-potency opioids were created, once again with the hypothesis that lower doses for pain would cause less addiction (as was initially assumed about heroin versus morphine). This effort led to the synthesis of the fully synthetic opioid fentanyl and its derivatives [1959, and US Food and Drug Administration (FDA) approved in 1968] (7). Contemporary efforts also aimed to develop opioids with only partial agonism or mixed agonism/antagonism for the subtypes of opioid receptor. The hope was that these partial agonists might be less rewarding and therefore less addictive. These efforts produced the synthetic drug loperamide (8) [1969, with FDA approval in 1976 as Immodium, an important antidiarrheal due to its activity in the gut (9)]. Loperamide is not addictive, but it is also nonanalgesic because it is rapidly transported out of the central nervous system by p-glycoprotein. Another semisynthetic opioid, buprenorphine (1969), was never FDA approved for pain treatment due to its poor analgesic ability compared to the other opioids, but it was approved as Suboxone and Subutex for treatment of OUD in 2002 (10).

With these successes at creating new opioids, but with little progress separating the analgesic effects from the addictive effects, the search for new opioid molecules subsided. The next significant opioid launch was simply a reformulation of oxycodone as a slow-release oral drug, which was FDA approved in 1995 without additional long-term tests. Purdue Pharma famously hailed OxyContin as nonaddictive, purporting that its slow release would not give a high. This false assumption is now credited for the inception of our current opioid crisis, when clinical practices shifted to a strictly pain-averse model, and unrestrained prescription access created a new generation of patients dependent on opioids.

This hiatus in opioid drug development and prelude to the US opioid crisis marked a highly productive time for basic research on opioids. This period saw the identification of the endogenous opioid peptides (1975–1977) (11–14) and the cloning of the four opioid receptors (1992–1994) (15–24), all of which are G protein–coupled receptors (GPCRs) of the G_i/o/z_ class. These discoveries enabled clarification of the precise mechanism of action of the existing opioid drugs. Important findings were that the analgesic effects of these drugs were mediated primarily by the mu-opioid receptor (MOR) (25) and all the drugs described above are agonists at the MOR. With a known GPCR target, a pharmacopeia of opioids, and a plethora of experimental approaches, the field was poised to return to the search for safer pain relief.

## SIGNALING AND REGULATION OF THE MU-OPIOID RECEPTOR

The analgesic action of opioids is dependent on MOR activation of trimeric G proteins. As a G_i/o_-coupled GPCR, the agonist-bound MOR promotes exchange of guanosine triphosphate (GTP) for guanosine diphosphate on G protein. The GTP-bound activated G protein inhibits adenylyl cyclase via the alpha subunit, which in turn decreases levels of cyclic adenosine monophosphate (cAMP) and the activity of protein kinase A. This receptor also inhibits neuronal activity through activation of GPCR inwardly rectifying potassium (GIRK) channels and inhibition of voltage-gated calcium channels (Ca_v_s). Opioids can thus hyperpolarize neurons through GIRKs and prevent transmitter release by reducing calcium influx through Ca_v_ inhibition. At the same time, they control levels of second messengers. These effects are all mediated by G protein. The adenylyl cyclase, GIRK, and Ca_v_ effects of opioids have been measured in many cell types in the central nervous system as well as in the gut and immune cells (26).

The strength and duration of this G protein signal are regulated by innate ligand properties such as off-rate and intrinsic efficacy but also by the rate of GTP hydrolysis, which can be increased through activity by a regulator of G protein signaling proteins (27). In addition, G protein signaling through the MOR, like that from most GPCRs, is regulated by a cascade of events that include direct phosphorylation of the MOR by GPCR kinases (GRKs) in response to ligand binding. Phosphorylation then facilitates the binding of arrestins. Arrestins, first discovered in 1986 as regulators of rhodopsin (28) and later of the beta-2-adrenergic GPCR (29), regulate signaling of most GPCRs. Arrestin recruitment to the receptor uncouples MOR from G protein, and scaffolds signal transduction by other second messengers including extracellular signal-regulated kinases and c-Jun N-terminal kinases. In addition, arrestin attracts a protein scaffold for internalization/endocytosis of the receptor. MORs that have been internalized by endocytosis are then dephosphorylated, deliganded, and recycled back to the cell surface for future activation (30).

Arrestin recruitment and desensitization and/or downregulation of MORs have received much interest as possible mechanisms of analgesic tolerance, which often results from repeated use of opioids. Because tolerance can necessitate dose escalation, increasing the risk for respiratory side effects and OUD, there was significant motivation to identify its underlying mechanisms. This has inspired many papers reporting changes in receptor quantity or measuring desensitization of receptor signaling during or following morphine treatment—both before and after arrestins were discovered and the MOR was cloned and could be expressed heterologously to isolate MOR-specific effects.

**Figure 2:**
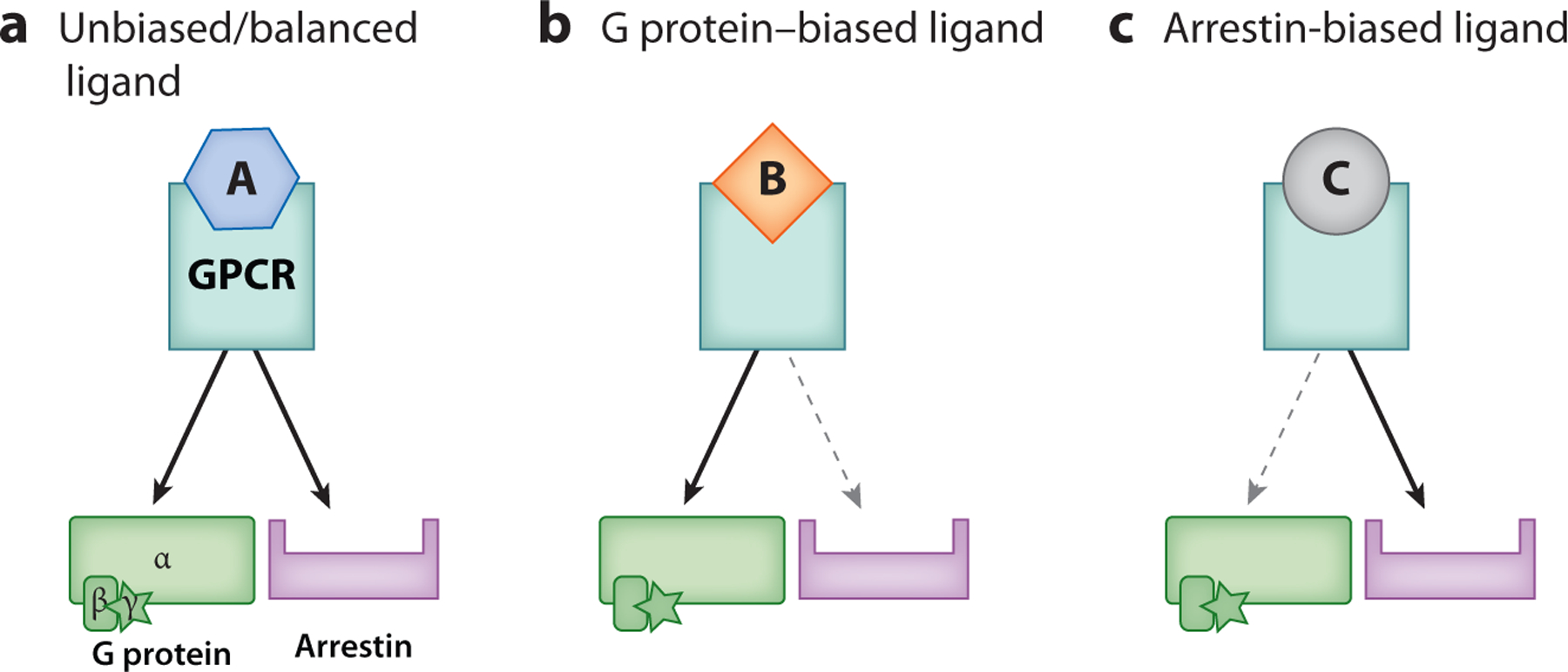
Schematic of balanced and biased agonism at an unspecified G protein–coupled receptor (GPCR). (*a*) An unbiased or balanced ligand (A) will cause the receptor to signal to both the G protein and arrestin pathways. (*b*) A ligand that is biased for G protein (B) will more effectively signal to the G protein pathway than to the arrestin pathway. (*c*) A ligand that is biased for arrestin (C) will more effectively signal to the arrestin pathway than to the G protein pathway. The arrow thickness indicates relative efficacy of signal compared to the other effector.

Distinct GPCR ligands can be differentially potent and/or efficacious at activating the G protein versus the arrestin signaling pathways (Figure 2). This functional selectivity for one GPCR effector versus another was first described by Roth & Chuang in 1987 (31) and has since been demonstrated for many classes of GPCR and coined signaling bias to reflect a gradient rather than a binary. The phenomenon of selective efficacy for one effector versus another received much skepticism until some second-generation antipsychotics were shown to promote serotonin 2A receptor (5-HT_2A_R) endocytosis despite being antagonists for G protein signaling (32). Bias became relevant to opioids in 1996 when it was found that, although the endogenous peptide agonists—and [d-Ala2, *N*-MePhe4, Gly-ol]-enkephalin (DAMGO), a hydrolysis-resistant enkephalin used as a surrogate for the endogenous ligand—promoted MOR endocytosis, morphine did not (33, 34). A series of subsequent studies demonstrated that this poor MOR endocytosis in response to morphine was due to low levels of GRK phosphorylation and arrestin recruitment at the MOR (35–37).

**Figure 3:**
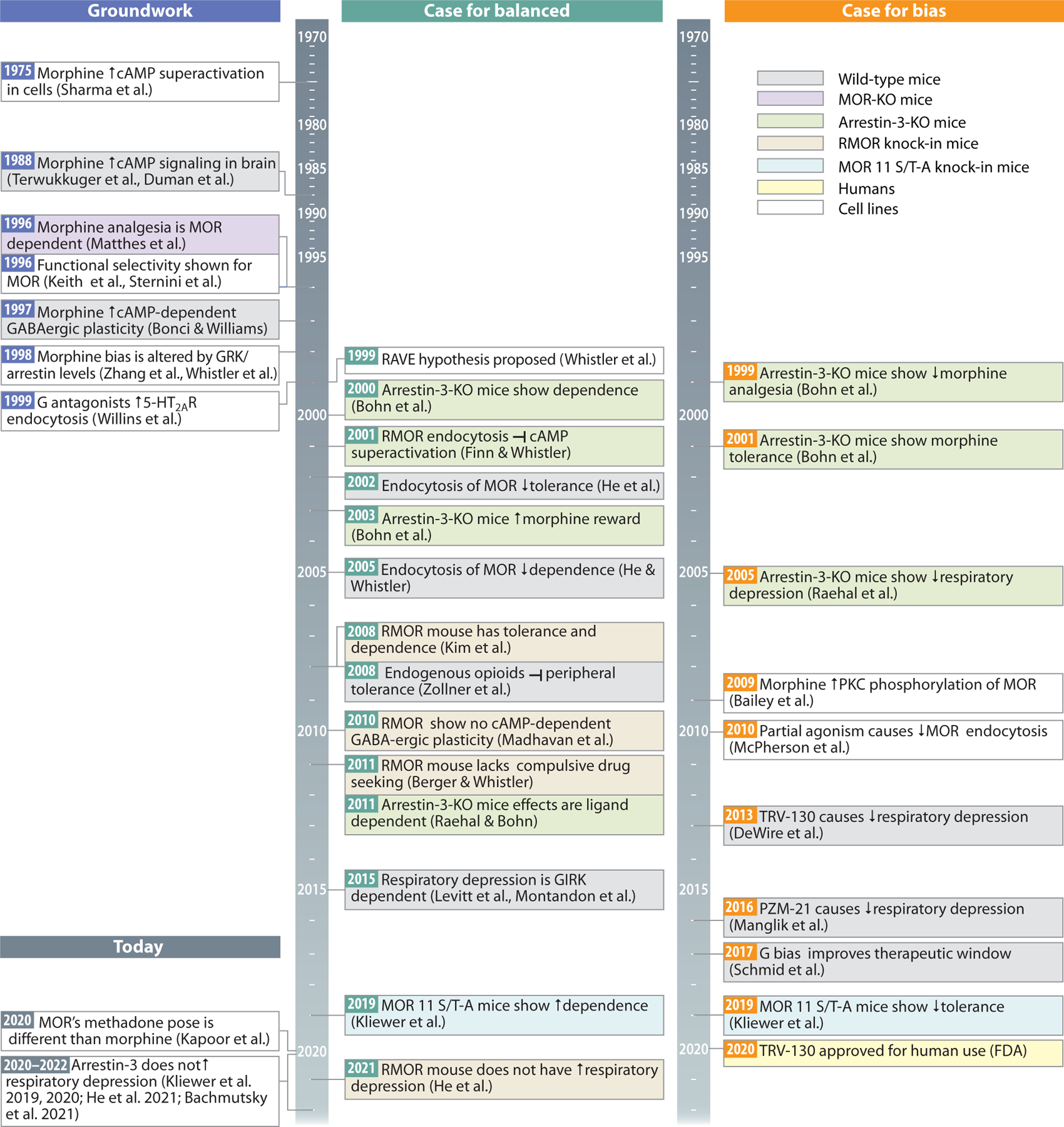
Historical timeline of key findings in support of balanced (*green*) versus biased (*orange*) opioid agonists. Hypotheses on the role of arrestin-3 diverged in 1999, and the field is now poised to begin a new era. Abbreviations: 5-HT_2A_R, serotonin 2A receptor; cAMP, cyclic adenosine monophosphate; FDA, Food and Drug Administration; GABA, gamma-aminobutyric acid; GIRK, GPCR inwardly rectifying potassium channel; GRK, G protein–coupled receptor kinase; KO, knockout; MOR, mu-opioid receptor; PKC, protein kinase C; RAVE, relative activity versus endocytosis; RMOR, recycling MOR; TRV-130, oliceridine.

The discovery that opioid drugs, but not opioid peptides, display signaling bias for G protein suggested a pathway and possible mechanism to separate the beneficial from the detrimental effects of opioids. Two competing hypotheses emerged from these results: that arrestins prevented the side effects and that arrestins were responsible for the side effects—a dichotomy that persists today (Figure 3). The role of signaling bias in the effect/side effect profiles of opioid drugs has been a focus of opioid drug development for more than two decades, with significant resources directed toward the dominant hypothesis that arrestin-3 activity is responsible for the negative side effects of opioid drugs. While this view has fueled the development of several new ultra-G-biased opioid compounds, its premise has been challenged. Some groups maintain the position that balanced agonists have more therapeutic potential. Others attribute effect/side effect profiles to drug properties other than bias.

**Figure 4:**
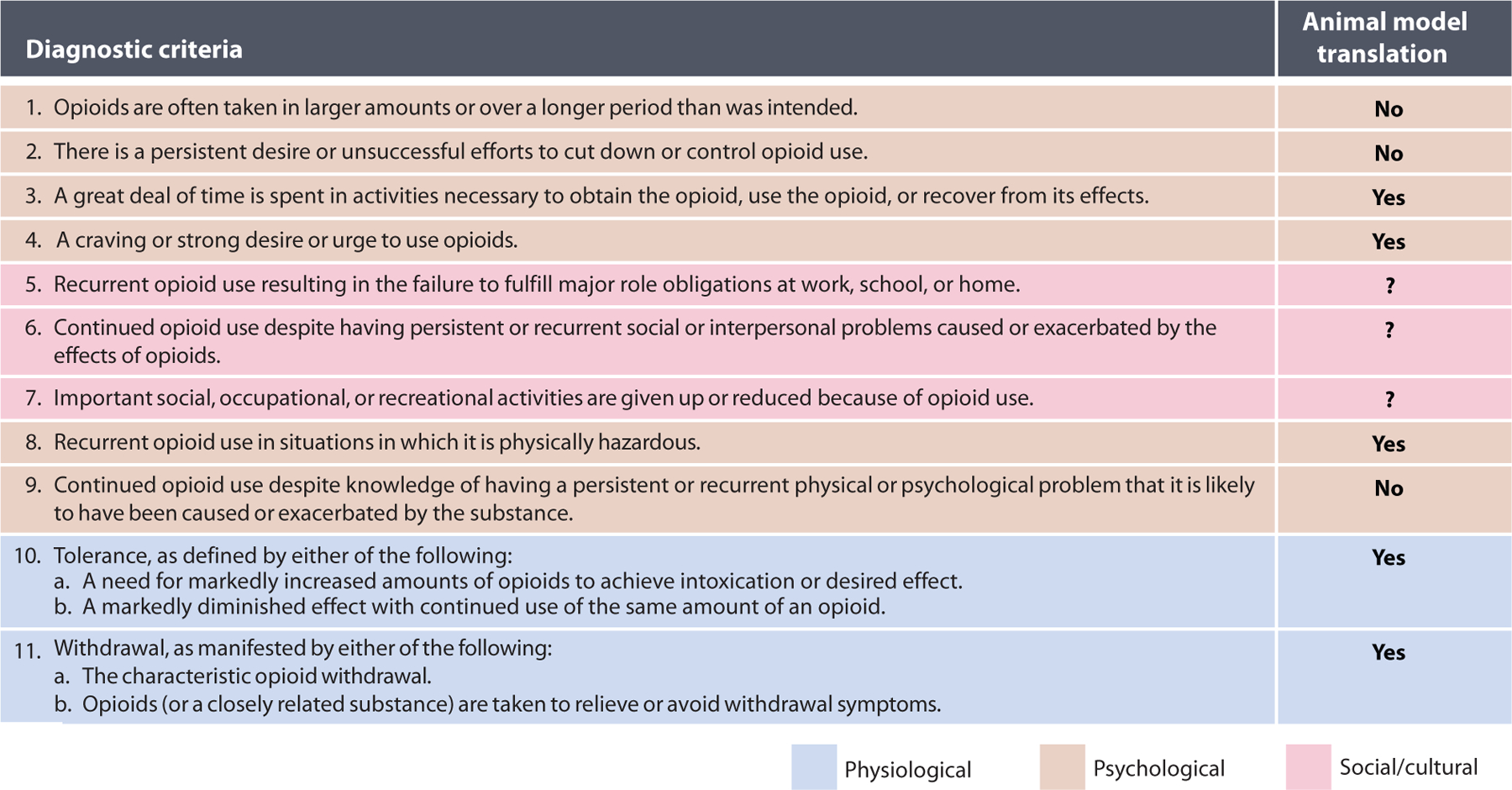
Diagnostic criteria for opioid use disorders (OUDs) as described in the *Diagnostic and Statistical Manual of Mental Disorders: DSM-5*^™^ (38). The term addiction is used colloquially to refer to OUD. Here, we classify each diagnostic criterion as belonging to one of three experiential categories and by our ability to observe or model it outside of humans. Symptoms that have physiological readouts (10, 11) have well-understood models. Some psychological symptoms (3, 4, 8) can be modeled by various drug seeking or self-administration paradigms. Symptoms that require self-evaluation or communication of intent by a patient (1, 2, 9) do not have available models. It is disputable whether the social or cultural components of OUD (5–7) can be reasonably modeled outside of humans.

Although the abuse potential of opioids is a side effect that garners much public attention, there are clear and meaningful limitations of discussing addiction in a basic research context. Substance use disorders, such as OUD, are complex human syndromes that present heterogeneously in the affected population. The *Diagnostic and Statistical Manual of Mental Disorders: DSM-5*^™^ (38), the current authority on psychiatric disease, defines OUD as two or more of eleven diagnostic criteria presenting within a twelve-month period (Figure 4). These criteria attempt to capture the range of the addiction experience, and OUD may be classified, based on how many criteria are met, as mild (2–3 symptoms), moderate (4–5 symptoms), or severe (6 or more symptoms) (38). Several of the *DSM-5* criteria rely on a degree of self-evaluation and/or must be evaluated within a human cultural context, which makes them difficult or impossible to evaluate in any model organism. Our discussion largely concerns the only two criteria on the list with direct physiological readouts: tolerance and withdrawal (dependence). We elaborate below on why mitigating these factors is particularly important when considering drug development end points. However, much work remains to connect the cellular processes described herein with their cognitive and behavioral correlates to unveil a more complete understanding of addiction biology.

In this review, we discuss the disputed relationship between arrestin-3 activity and opioid side effects and consider the complexities of pharmacology beyond bias. Furthermore, we attempt to reconcile disparate claims about the role of signaling and signaling bias in drug effect profiles and therapeutic windows in a way that takes all the available data into account. It is our hope that the reconciliation of these claims might inform future directions of research and drug development.

## A BIASED VIEW OF OPIOIDS

A key moment in opioid signaling bias research came with the observation that arrestin-3 knockout mice responded differently to morphine than their wild-type counterparts with potentiated analgesia and reduced tolerance, among other effects (39–41). This led to the hypothesis that receptor desensitization, which is regularly portrayed as the main source of tolerance in response to drugs, is mediated by phosphorylation and arrestin-3 recruitment to the MOR. Shortly thereafter, the same group demonstrated that morphine-induced respiratory suppression was attenuated by germline knockout of arrestin-3 (42). This observation became the bedrock for a drug discovery strategy that prioritized the design of ultra-G-biased agonists to the MOR that promoted no arrestin-3 recruitment with the goal of mimicking the result of its genetic elimination. This occurred without independent replication of the result or further understanding of the mechanism behind the altered respiratory depression. In the last two decades, significant resources have been put behind this cause, leading to the development of a few novel ultra-G biased agonists. One, oliceridine (TRV-130), has recently received approval for clinical use (43, 44). The primary goal of this research was to circumvent opioid-induced respiratory depression (OIRD), the cause of opioid overdose deaths.

Despite the early studies of arrestin-3 knockout mice that inspired a generation of research seeking to eliminate arrestin-3 activity at the MOR, the original respiratory results have proven difficult to replicate. A consortium of three independent laboratories across the world has reported intact morphine-induced respiratory depression in these mice (45), consistent with what our laboratory (46) and another (47) have observed. We sought to elucidate the mechanism that explains these differences and have postulated that the mixed genetic background of the original arrestin-3 knockout mice is likely a source of their resistance to OIRD (46). Improving upon the resolution offered by a germline knockout of arrestin-3, which undoubtedly alters signaling from receptors other than MOR, one group has also found no resistance to OIRD in a knock-in mouse that is incapable of recruiting arrestin-3 to the MOR due to substitution of all residues at key phosphorylation sites in the c-tail (MOR 11S/T-A) (48).

Additionally, our lab found that a panel of clinically relevant opioid analgesics with varying degrees of signaling bias all promoted respiratory depression at equianalgesic doses in wild-type animals, with observable differences in the timing, but not the severity, of this effect (46). A notable exception was buprenorphine, which produced little respiratory depression at equianalgesic doses. Although buprenorphine is not used clinically as an analgesic, it is worth exploring whether this result represents a buprenorphine-specific signaling mechanism from the MOR or its activity at targets other than the MOR (such as antagonism of the kappa-opioid receptor or activity at the nociceptin receptor). When imagining new opioid ligands, it is intriguing to consider that agonism at the nociceptin receptor can attenuate the rewarding effects of opioids (49). This body of evidence presents a compelling case against arrestin-3 engagement at the MOR as the cause of OIRD. There is, however, recent work suggesting that morphine may cause additional respiratory depression in comparison to DAMGO (50), highlighting the need for more comprehensive respiratory studies exploring how endogenous and exogenous opioids contribute to OIRD in order to probe any role of bias that the aforementioned studies have missed. In the meantime, we believe that ample evidence contradicting the foundational result upon which contemporary drug development strategies were based is grounds to reevaluate these strategies and broaden the search for mechanisms of interest.

## EXPLOITING ENDOGENOUS MECHANISMS FOR IMPROVED OUTCOMES: DOES NATURE KNOW BEST?

**Figure 5:**
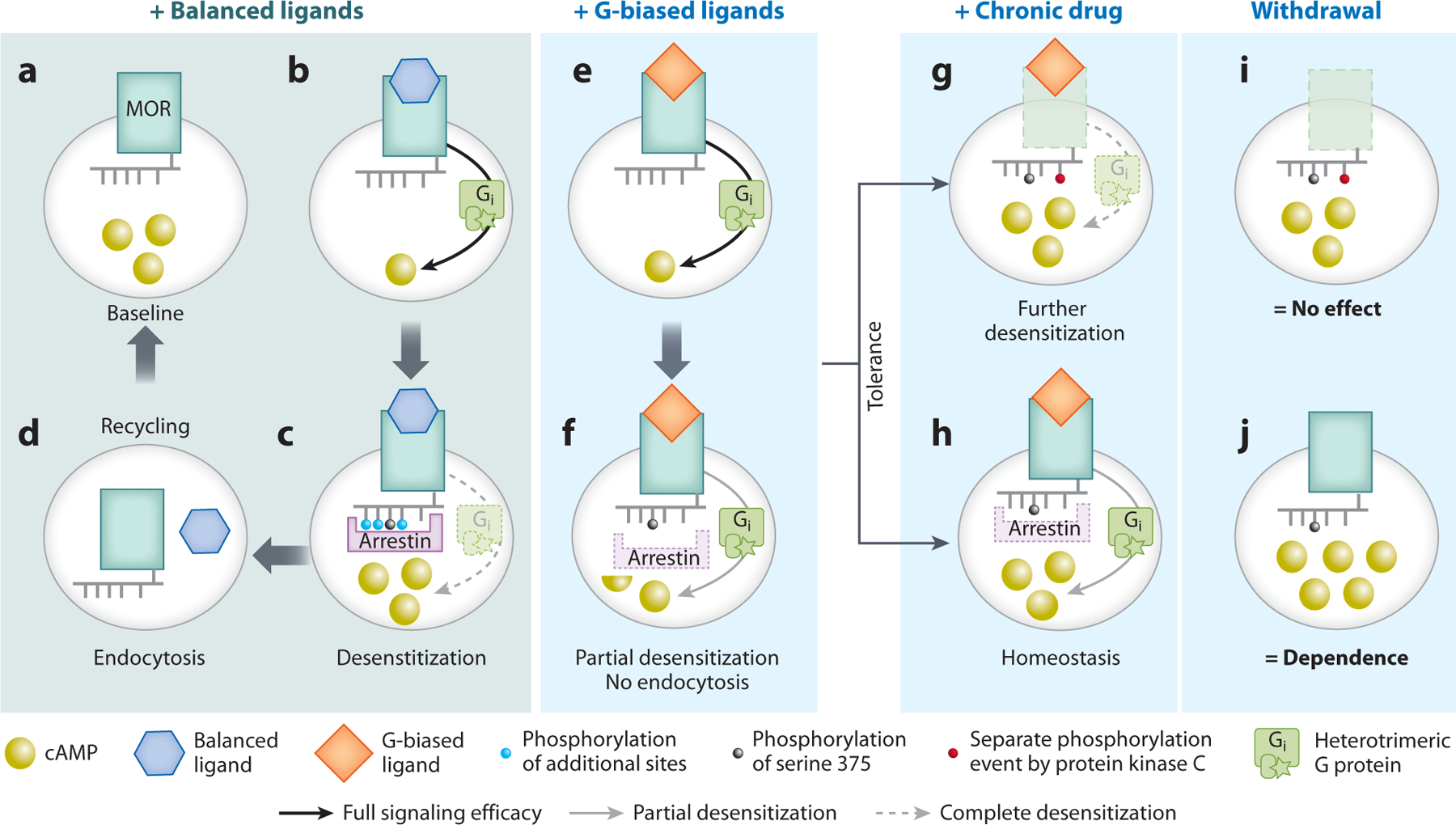
Schematic of opioid tolerance produced via desensitization versus cellular homeostasis. (*a*–*d*) Signaling response of MOR to balanced ligand (*blue hexagons*). (*a*) Empty receptor and baseline levels of cAMP (*yellow spheres*). (*b*) Balanced agonist at MOR promotes signaling to G_i_, decreasing cAMP compared to panel *a*. (*c*) Complete phosphorylation of MOR (*gray* and *light blue spheres*) and strong arrestin recruitment lead to rapid desensitization of the G protein signal and return to baseline cAMP levels. (*d*) Endocytosis and recycling of MOR lead to rapid resensitization. (*e*–*j*) Signaling response of MOR to biased ligand (e.g., morphine; *orange diamonds*). (*e*) Acute morphine promotes signaling to G_i_, decreasing cAMP compared to an empty receptor (panel *a*). (*f*) Single phosphorylation of MOR on serine 375 (*gray spheres*) and poor arrestin recruitment lead to weak desensitization of G protein. Once receptors are phosphorylated and partially desensitized, they remain this way without endocytosis and resensitization. (*g*,*h*) Chronic morphine produces tolerance by two mechanistically distinct processes. (*g*) Tolerance via further receptor desensitization. PKC phosphorylates MOR (*red spheres*), further uncoupling it from G protein and leading to higher cAMP levels (*yellow spheres*) in the presence of morphine compared to acute morphine shown in panels *e* and *f*. (*h*) Tolerance via homeostasis. Cells increase cAMP levels by mechanisms independent of MOR signal (e.g., increased adenylyl cyclase, decreased cAMP phosphodiesterase, increased signaling via G_s_-coupled receptors). This also leads to higher cAMP levels (*yellow spheres*) in the presence of morphine compared to acute morphine shown in panels *e* and *f*. Tolerance via further desensitization (*g*) and tolerance via a homeostatic shift (*h*) are therefore indistinguishable in the presence of morphine. (*i*,*j*) Withdrawal following tolerance by the two mechanisms shown in panels *g* and *h*. (*i*) Withdrawal of morphine has no effect on cAMP levels when tolerance is produced only by receptor desensitization because the receptors are not functional. cAMP levels are the same as baseline seen in panel *a*. (*j*) Withdrawal of morphine causes an increase in cAMP (*yellow spheres*) to superactivation levels above those at baseline in panel *a*, revealing both the presence of the homeostatic shift and that MORs are still functional and controlling levels of cAMP. This cAMP superactivation manifests as withdrawal signs of dependence. Abbreviations: cAMP, cyclic adenosine monophosphate; MOR, mu-opioid receptor; PKC, protein kinase C.

While much of the field championed the pursuit of ultrabiased agonists, our group has pursued an opposing story: how enhanced arrestin-3 recruitment to the MOR alters signaling and in vivo responses to opioids. Inspired by the observation that the endogenous ligands all engage arrestins but do not produce tolerance under conditions when exogenous drugs do, in 1999 we proposed the RAVE hypothesis: that Relative Activity (at G protein) Versus amount of Endocytosis (in effect, signaling bias) would be predictive of tolerance and dependence to opioids. In this classification, endogenous peptide ligands have a low RAVE because G protein signal is titrated by arrestin/endocytosis, while the opioid drugs have a high RAVE because G protein signal is not opposed by arrestins. Our hypothesis was that ligands with a high RAVE would cause homeostatic adaptations to oppose MOR signaling and that these would manifest as tolerance in the presence of drug and dependence upon withdrawal of drug (Figure 5g–j). Simply put, chronic signaling through G_i_, untitrated by arrestin-3, would demand a rebalancing to homeostasis. One such homeostatic adaptation is cAMP superactivation, a compensatory increase in cAMP levels following prolonged adenylyl cyclase inhibition, a well-established cellular hallmark of morphine tolerance (51–55).

Shortly after we proposed that high RAVE would produce tolerance (and dependence), arrestin-3 knockout mice were shown to have enhanced analgesia and reduced analgesic tolerance to morphine, emboldening efforts to demonstrate that desensitization of MORs alone creates tolerance. Importantly, these are not mutually exclusive mechanisms (Figure 5) and could occur simultaneously, either in the same cells, or possibly separated by cell type.

To understand the relationship between trafficking of the MOR and the effects of opioids, we coadministered DAMGO with morphine in wild-type rats and found it prevented analgesic tolerance (56). We hypothesized that this was due to homodimerization of the MOR wherein one receptor occupied by DAMGO is sufficient to recruit arrestins to the morphine-occupied receptors. The MOR has since been shown to dimerize in a ligand-dependent manner (57). We expanded these studies using methadone (58), the only FDA-approved opioid analgesic that approaches the balanced signaling of the endogenous peptides (59), in hopes of creating an achievable therapeutic strategy. Rats given a cocktail of morphine spiked with methadone at doses that provided no additional analgesia do not develop tolerance or dependence, an effect we showed was independent of methadone’s activity at the *N*-methyl-d-aspartate (NMDA) receptor (58). Histology demonstrated that this dual opioid cocktail promoted endocytosis of the MOR, whereas neither morphine, nor the subanalgesic dose of methadone used in the study, were sufficient to do this on their own (58). These results complement those suggesting that some pain patients are protected from tolerance and dependence to exogenous opioids due to naturally elevated levels of endorphins and enkephalins. This has been shown in rodent models of tolerance during inflammatory pain (60).

Given the lack of drug-like ligands with which to further interrogate the downstream effects of balanced opioid signaling, we turned to a genetic approach with the development of RMOR (for recycling mu-opioid receptor). RMOR is a chimeric receptor containing a 22-amino-acid substitution in the cytoplasmic tail with a sequence from the closely related delta-opioid receptor (DOR). This substitution gives enhanced arrestin-3 binding capacity while the G protein signaling is unchanged. In effect, signaling and trafficking of the RMOR respond to morphine much like the wild-type MOR responds to DAMGO (61). While creation of this receptor was done stochastically, we now know this sequence replaced the phosphorylation bar code of the MOR (62) with that of the DOR. This makes RMOR a better substrate for GRKs so it is more highly phosphorylated when bound to morphine, thereby facilitating arrestin-3 recruitment (35). The phosphorylation barcode for robust arrestin-3 recruitment was carefully interrogated in 2018 (62). Briefly, this group demonstrated that the wild-type MOR is phosphorylated on four distinct residues in response to DAMGO, whereas morphine-occupied MORs are phosphorylated on only one of these (S375), unless GRKs are highly overexpressed.

With this RMOR tool in hand, we could pursue our hypothesis without the caveats inherent to comparing ligands that differ in other pharmacological properties beyond bias. In cell-based assays, we demonstrated that cells expressing the MOR but not RMOR show cAMP superactivation, a key component of tolerance and dependence (61). We then created a knock-in mouse expressing the RMOR. In 2008, we reported that RMOR mice were highly resistant to morphine tolerance after repeated dosing and did not exhibit withdrawal behaviors precipitated by naloxone (63). We then demonstrated in the RMOR mouse model that adaptations following chronic morphine are prevented when signaling is altered in the direction of the endogenous ligands (64). This includes cAMP superactivation, which is necessary for withdrawal behaviors (65–68). We had previously shown that methadone and DAMGO promote reduced cAMP superactivation compared to morphine (56, 61). This supported our hypothesis that balanced signaling and MOR recycling impede the cellular conditions that lead to tolerance and dependence. In slice electrophysiology studies, we found that the ventral tegmental area (VTA) dopamine neurons of RMOR mice were not subject to potentiated inhibition (65), a form of homeostatic plasticity that appears during opioid withdrawal (69–71). Mice lacking arrestin-3 also show potentiated inhibition, even when opioid drug naïve, suggesting that endogenous ligands can cause similar plasticity as morphine when MORs are unable to engage arrestins (72). Finally, despite potentiated analgesia and reward in response to morphine, RMOR mice do not transition to a pattern of compulsive drug taking behavior in a complex operant administration model of OUD (73), indicating a promising connection between receptor trafficking and abuse liability.

The pharmacokinetics of morphine—as well as any off-target effects—are unaltered in RMOR mice, and RMOR and wild-type mice have the same number of opioid receptors (63). The efficacies of morphine and DAMGO are equivalent for both activation of GIRK channels and inhibition of transmitter release in the VTA (65). Furthermore, the 22-amino-acid substitution is entirely contained within exon 3, meaning any putative MOR splice variants (74) that alter their endocytosis (75) also carry this new GRK barcode. Although we cannot rule out that the RMOR, but not the wild-type MOR, signals to an unidentified effector specific to DORs to protect against tolerance, dependence, and compulsive drug seeking, this seems unlikely, as deletion of the DOR actually reduces tolerance to morphine (76). Also, while morphine analgesia (63) and reward (73) are enhanced in RMOR mice compared to wild-type mice, methadone analgesia and reward are indistinguishable. All of these data indicate that the change in signaling bias with morphine in RMORs, rather than a change in general RMOR signaling, is responsible for the reduced tolerance, dependence, and compulsive drug seeking in RMOR mice.

These findings arose during a period of opioid history when elimination of arrestin-3 activity in order to reduce side effects was the dominant hypothesis and drug development strategy (Figure 2). As mentioned above, the goal at the time was to ameliorate OIRD with an ultra-G-biased signaling profile. If OIRD was indeed a direct result of engaging the arrestin-3 pathway, one would expect RMOR mice, with their enhanced arrestin-3 recruitment, to have exacerbated respiratory suppression on opioid drugs. However when we tested this, we found that OIRD was slightly exacerbated in arrestin-3 knockout mice compared to wild-type mice (46). RMOR mice had a respiratory response indistinguishable from wild-type mice, strengthening the hypothesis that arrestin-3 activity is not causal for OIRD. As the previously favored hypothesis that arrestin-3 engagement produces respiratory depression has now been broadly overturned, and OIRD is widely believed to result from G protein activity (77, 78) (inseparable from analgesia), now is an ideal time to reexamine the implications of the RMOR results. From our perspective, this means prioritizing signaling that is balanced in accordance with the endogenous ligands as a possible avenue for reducing critical side effects including tolerance, dependence, and abuse liability. This would be unprecedented, and as mentioned previously, methadone is the only clinically utilized opioid with a signaling profile similar to the endogenous ligands, though it is rarely used as a first-line analgesic. However, in the few human studies where methadone and morphine were compared in opioid naïve pain patients (patients who had not undergone morphine-induced changes in plasticity causing tolerance and/or dependence), methadone showed less tolerance and less severe withdrawal (79).

## BARRIERS TO CONSENSUS: THE SPECIFICS AND SEMANTICS OF PHARMACOLOGICAL BIAS

The stakes of this topic are professionally, financially, and ethically high, so it is unsurprising that the climate around this work, and the discussion of bias in particular, has grown contentious as contradictory results come to light. Thoughtful research is needed to address the apparent incompatibility of results across laboratories in order to move toward a common understanding.

At the core of this controversy is the debate over whether bias for G protein is or is not the explanation for side effect reduction, as some studies claim (42, 43, 80, 81) and others refute (45, 46, 48, 82, 83). The present trend is to explain conflicting results through differences in how bias is quantified (84–86). One competing hypothesis that has gained recent momentum is that side effects are driven by the intrinsic efficacy of opioid agonists (85) and that the correlation of G protein activity and arrestin-3 recruitment (87) has led to the misattribution of these effects to arrestin-3 activity. While this may be true, some supporting experiments involved the overexpression of GRKs, which alters the efficacy of arrestin-3 recruitment and, by most definitions, would change bias as well. Overall, we have arrived at an effective stalemate in the literature in which each group claims that they are properly determining bias, a metric with no consensus-based standard.

Pitfalls of bias calculation strategies have been extensively reviewed (84, 88–90), so we do not provide further analysis on the available models. Importantly, signaling bias is inherently a relative measure, so it is challenging to compare the bias levels of various compounds across studies. Bias quantification requires that a separate dose response curve be determined empirically for each different effector, typically G protein and arrestin-3. This requires a unique assay for each effector, and the options available are favored or avoided for various reasons. These assays employ artificial systems, often with some degree of signal amplification. Additionally, because no assay is widely accepted as the standard, comparing results across methods is difficult. Once dose-response curves are generated, the relative activity of these two effectors must be compared to that elicited by a reference compound, the choice of which is critical for the intended impact of the study. This raises another challenge in comparing work across groups due to differing opinions on which reference compound will generate the most relevant bias calculation. When the reference compound is a peptide, as is often the case in opioid studies, its potency is particularly vulnerable to factors like storage and preparation. Because experimental methods and common-use definitions vary between labs, the same drug can easily be classified as biased in one case and unbiased in another. Beyond procedural challenges, real biological phenomena can also complicate how bias is appreciated. For example, in different cellular compartments within a single neuron there are differences in the ability of morphine to promote receptor endocytosis (91).

Because methods of bias calculation all suffer their own flaws and are therefore selected based on opinion and preference, we fear that these arguments lack a clear end point. Rather than pursue the debate on how best to calculate bias, we suggest it would be more valuable to return to the original question of the role of bias in opioid side effects. One avenue that has been underexplored is how side effects are influenced by agonism that is balanced or arrestin-biased. Following the canonical arrestin-3 knockout results, we know of no lab other than our own that has explored balanced agonism in earnest, even for the purpose of supporting the hypothesis that biased agonists perform better. This is made more difficult by the paucity of balanced agonists, although methadone, a full agonist at G protein that robustly recruits arrestin-3 even without GRK overexpression, has been excluded from many of the analyses.

The recent review from Kolb et al. (88) gives an excellent explanation of how bias can be defined relative to any compound of interest (benchmark bias), a physiologically dominant agonist (physiological bias), or a GPCR signaling equally to both effectors (pathway bias). In translational studies of opioids, our group is most concerned with physiological bias (bias measured relative to an endogenous agonist such as an endorphin or enkephalin). This practice is common enough but has largely been employed with the specific motive of identifying molecules that are unlike the endogenous agonists in their signaling behaviors. It is likely that bias of any opioid varies across tissue and cell type, not just because levels of GRKs and arrestins vary, but because efficacy does too. For example, neurons that are tonically active will look more sensitive to inhibition by opioids than neurons that rarely fire. Our view is that in this new chapter of opioid research we should instead cast more light on how unbiased, or balanced, signaling could be an avenue to reduce the negative impacts of opioid use. We see this as the appropriate angle from which to approach drug development because, while signaling activity must be measured in vitro, at least if we wish to screen many compounds, these drugs are ultimately intended for use in a complex organism. The system in which opioid drugs are intended to operate is calibrated to the signaling profile produced by its own endogenous agonists. It follows that a drug with a similar signaling profile could exploit the endogenous analgesia mechanism while minimally perturbing the state of homeostasis.

**Figure 6:**
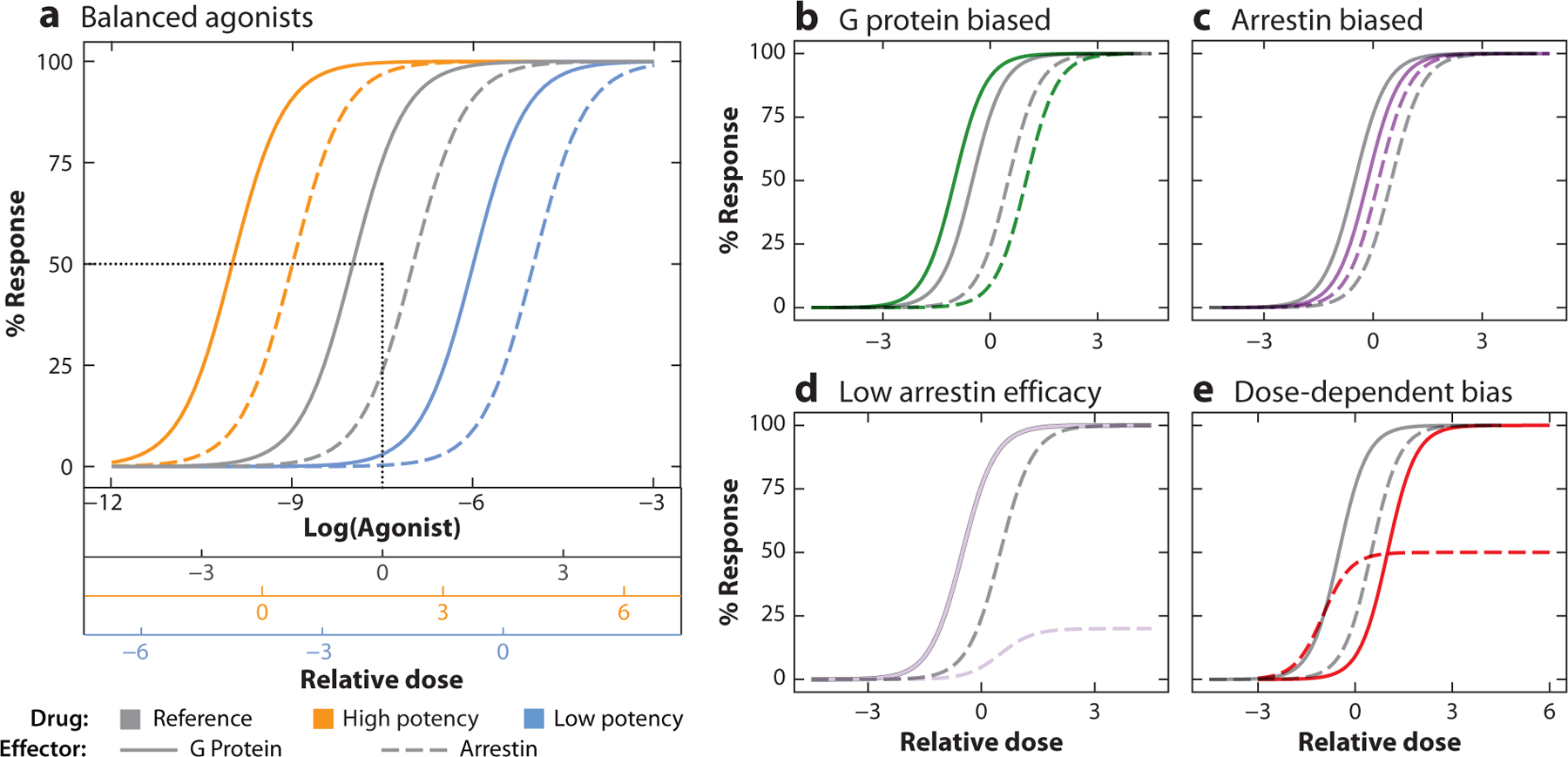
Examples of G protein and arrestin signaling profiles for balanced and biased agonists. (*a*) Agonists of low (*blue*) and high (*orange*) potency can be balanced when their G protein (*solid lines*) and arrestin (*dashed lines*) dose response curves are equivalently shifted from those of the reference compound (*gray*). Each agonist has a relative *x* axis determined by setting the midpoint between its two EC50 values as zero (demonstrated for the reference agonist with *black dotted lines*). Fully balanced agonists would therefore show superimposed curves if the *x* axes were aligned at their relative doses of zero. (*b*–*d*) Example drugs (*colors*) aligned to the reference compound (*gray*) at relative dose zero. (*b*) A G protein–biased drug will have a G protein curve that is more left shifted from its arrestin curve than the reference compound. (*c*) An arrestin-biased drug will have a G protein curve that is less left shifted from its arrestin curve than the reference compound. (*d*) A drug can be G protein biased if it is a partial agonist for arrestin, even when potency for both G and arrestin are identical to the reference compound (G protein curves are superimposed for the two compounds). (*e*) A drug that is more potent but less efficacious for arrestin recruitment will be arrestin biased at lower doses and G biased at higher doses.

As we explain in Figure 6, we consider signaling to be balanced when the relationship between G protein and arrestin-3 is equal to that of the reference compound at the same relative dose. It is therefore possible for agonists of variable potencies to be balanced provided the relationship of the two effectors reflects that of the reference compound (Figure 6a). If both the G protein and arrestin dose response curves are shifted the same amount relative to the reference curves, the compound is considered balanced. Bias can occur via a change in either potency or efficacy at either effector (Figure 6b–e). This also means that it is theoretically possible for an agonist to be balanced at some doses and biased at others, or even G protein biased at some doses and arrestin biased at others (Figure 6e). This is not merely hypothetical, as some second-generation antipsychotics are arrestin biased, doing a better job of promoting endocytosis of their target receptors (32) and/or engaging arrestin-mediated signaling there (92).

This esteem for balanced agonism is not merely philosophical; it is well supported by several studies, as described above. However, we need more studies and more balanced ligands to thoroughly test this hypothesis. This does not appear to be an impossible task. Most of the more recent ligands discovered have been ultrabiased because that was the intended product (TRV-130) or possibly because the structure used for virtual screening was not suited to identify balanced ligands, not because balanced ligands do not exist. It was recently shown using molecular dynamics simulations that the conformations displayed by a methadone-occupied MOR are distinct from those for a morphine- or TRV-130-occupied receptor (93), indicating that a different structure might identify additional balanced MOR ligands. A deeper dive into the literature shows that even in chemical series designed to identify ultra-G-biased ligands, such as the herkinorins and recent SR series, more balanced ligands were identified (see compound 7B in Reference 94 and SR-14969 in Reference 80). Tianeptine, an antidepressant whose activity is mediated through MOR, may also be more balanced than existing opioids and shows reduced tolerance and dependence (95). These hints, coupled with a recent natural products library screen that identified a balanced, albeit low potency MOR agonist (96), suggest that novel balanced opioids are within reach.

## MOVING FORWARD WITH A RECONCILED VIEW OF CONTRADICTORY RESULTS

**Figure 7:**
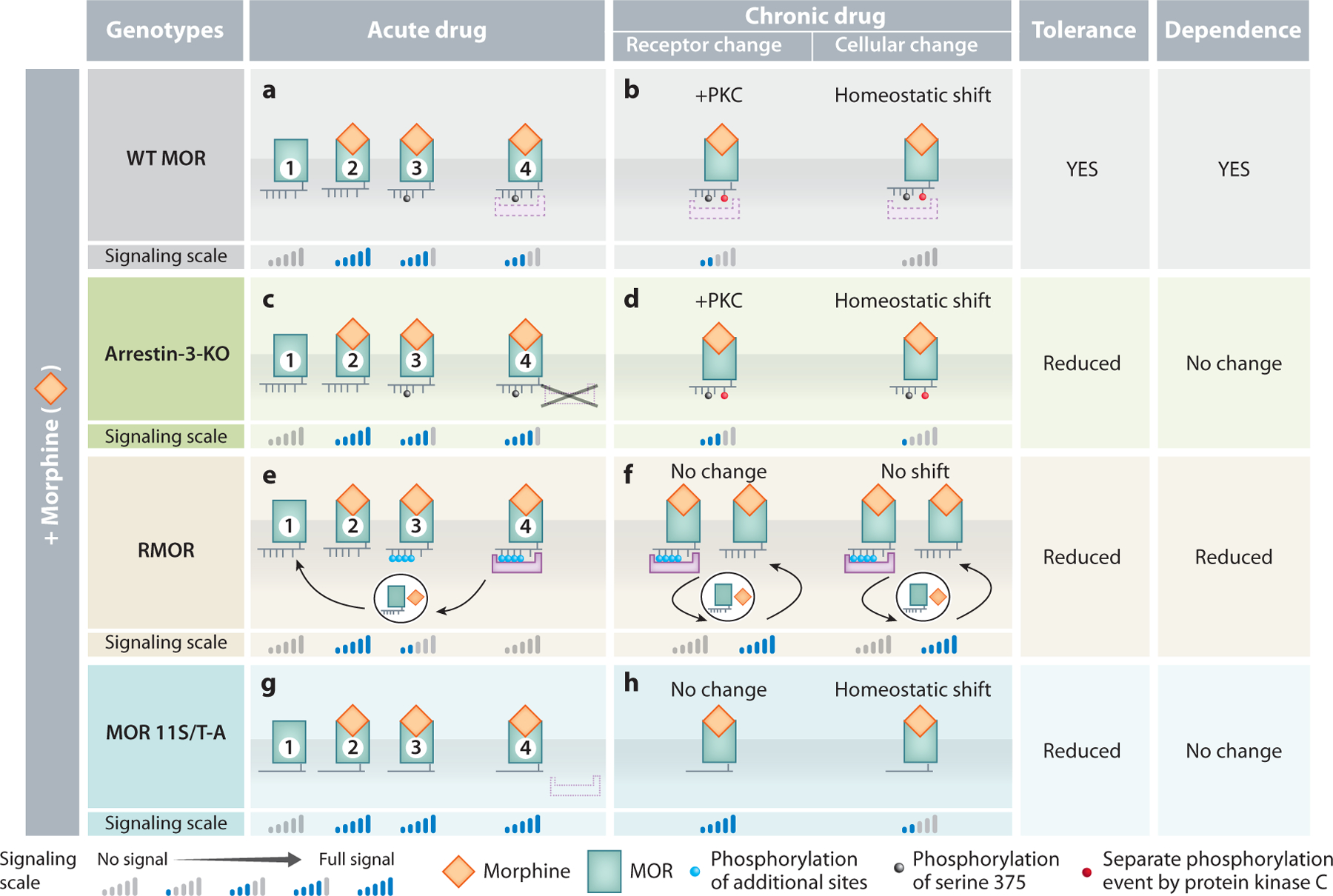
Model of desensitization mechanisms and cellular homeostatic shift in response to acute and chronic morphine in four genotypes of mice: WT, arrestin-3-KO, RMOR knock-in, and MOR 11S/T-A knock-in. (*a*,*b*) Signaling cycle of the WT MOR in response to acute morphine. (*a*) Morphine-occupied MOR is phosphorylated only on serine 375 (S375) (③), partially reducing signal. The partially phosphorylated MOR weakly recruits arrestin-3 (④), further reducing but not eliminating signal. This weak arrestin-3 recruitment is not sufficient to promote endocytosis and recycling so weakened receptor signaling persists. (*b*) Chronic morphine. The persistent signal from partially phosphorylated MORs triggers PKC phosphorylation, which further reduces (but does not eliminate) signaling. Homeostatic adaptations compensate further for persistent signaling. Both PKC phosphorylation and the homeostatic shift contribute to tolerance. (*c*) WT MOR response to acute morphine in arrestin-3-KO mice. As in WT mice (*a*), MOR is phosphorylated only on S375 (③), reducing signal, but no further reduction occurs via arrestin-3 (④). This explains the enhanced acute analgesia with morphine in arrestin-3-KO mice compared to WT. (*d*) Chronic morphine. The persistent signaling promotes PKC phosphorylation, further reducing signal. Homeostatic adaptations also compensate for the persistent signal, but because the receptors are more active due to no arrestin-3-mediated desensitization, tolerance is reduced compared to WT mice. (*e*) Signaling cycle of the RMOR in response to acute morphine. Morphine-occupied RMOR (②) is completely phosphorylated (③), desensitizing signal. Arrestin-3 recruitment completes the desensitization (④), halting signaling and promoting endocytosis, recycling, and signaling restoration in response to ligand, initiating another cascade. Receptor recycling prevents the partial desensitization that occurs in WT, which explains the enhanced morphine analgesia in RMOR mice. (*g*) Signaling cycle of the MOR 11S/T-A in response to acute morphine. MOR 11S/T-A will not be phosphorylated (③) nor recruit arrestin (④). This explains the enhanced analgesia compared to WT mice. (h) Chronic morphine. The persistent signaling will promote homeostatic adaptations reducing signal and causing tolerance, but because receptors are more active due to no GRK or arrestin-3 desensitization, there will be less tolerance than WT mice. Abbreviations: GRK, G protein–coupled receptor kinase; KO, knockout; MOR, mu-opioid receptor; PKC, protein kinase C; RMOR, recycling MOR; WT, wild-type.

The relationship between MOR trafficking, tolerance, and dependence (97) has been overshadowed by the focus on defining signaling bias and reducing respiratory phenotypes. Tolerance is a highly consequential side effect of opioids given its underlying role in both dose escalation and dependence, common precursors to addiction. A responsible drug development strategy will therefore direct special scrutiny toward tolerance outcomes. We define tolerance as a diminished response to a drug following previous exposure (38) (Figure 4), a broad definition to encompass the myriad mechanisms that could be responsible for this effect. In a GPCR-mediated drug response, tolerance can be caused by changes to the receptors themselves or changes independent of receptors that occur prior to or downstream of agonist binding (Figure 5g,h). As discussed above, both increasing engagement with arrestins (in RMOR mice) and decreasing engagement with arrestins (in arrestin-3 knockout mice and MOR 11S/T-A knock-in mice) enhance analgesia and reduce tolerance. It is in the best interest of the research community and the public to explain how both things can be true. Here, we propose a model wherein morphine tolerance is mediated both by partial desensitization of MORs and by homeostatic adaptations to prolonged G protein signaling that is poorly titrated by endocytosis and recycling (Figure 7). We favor this model because it reconciles the observations made in wild-type, arrestin-3 knockout, MOR 11S/T-A, and RMOR mice.

For many drugs, including opioids, it is common practice to treat receptor desensitization as a surrogate for tolerance. Many distinct mechanisms can cause desensitization of a receptor. In canonical GPCR signaling, c-tail phosphorylation partially disrupts the strength with which the receptor couples to G protein. Recruitment of arrestins to these phosphorylated receptors causes more pronounced desensitization as G protein coupling is further impeded (98), and the process is completed by removal of the receptors from the surface via endocytosis. Following endocytosis, GPCRs are either recycled to the plasma membrane (resensitized) or targeted to the lysosome for degradation (downregulated). After desensitization and endocytosis, MORs are recycled to the plasma membrane, not degraded, and thereby resensitized (99), a process that appears to be altered following chronic morphine but not chronic methadone treatment (100). Desensitization prevents receptors from initiating their signaling cascade and requires more drug to increase the number of occupied receptors, thus compensating for those rendered ineffective. Tolerance caused by desensitization is therefore similar to tolerance due to a reduction in actual receptor number caused by receptor degradation/downregulation, not a typical fate of activated MORs (99). Desensitization of the MOR is variable across cell and tissue types, dependent on GRK and arrestin expression levels, and can differ based on which effector is measured (101). For example, GIRK activation by the MOR in the periaqueductal gray is desensitized by enkephalins, while MOR inhibition of transmitter release is not (102). In these presynaptic terminals, MORs are still endocytosed following DAMGO treatment (103), indicating that the desensitization machinery remains intact. This does not translate to a change in apparent efficacy, presumably because there are enough spare receptors to amplify the signal. Functional response to morphine can also change at the cellular level even with no change in efficacy for G protein activation, a nonamplified signal (104).

**Figure 8:**
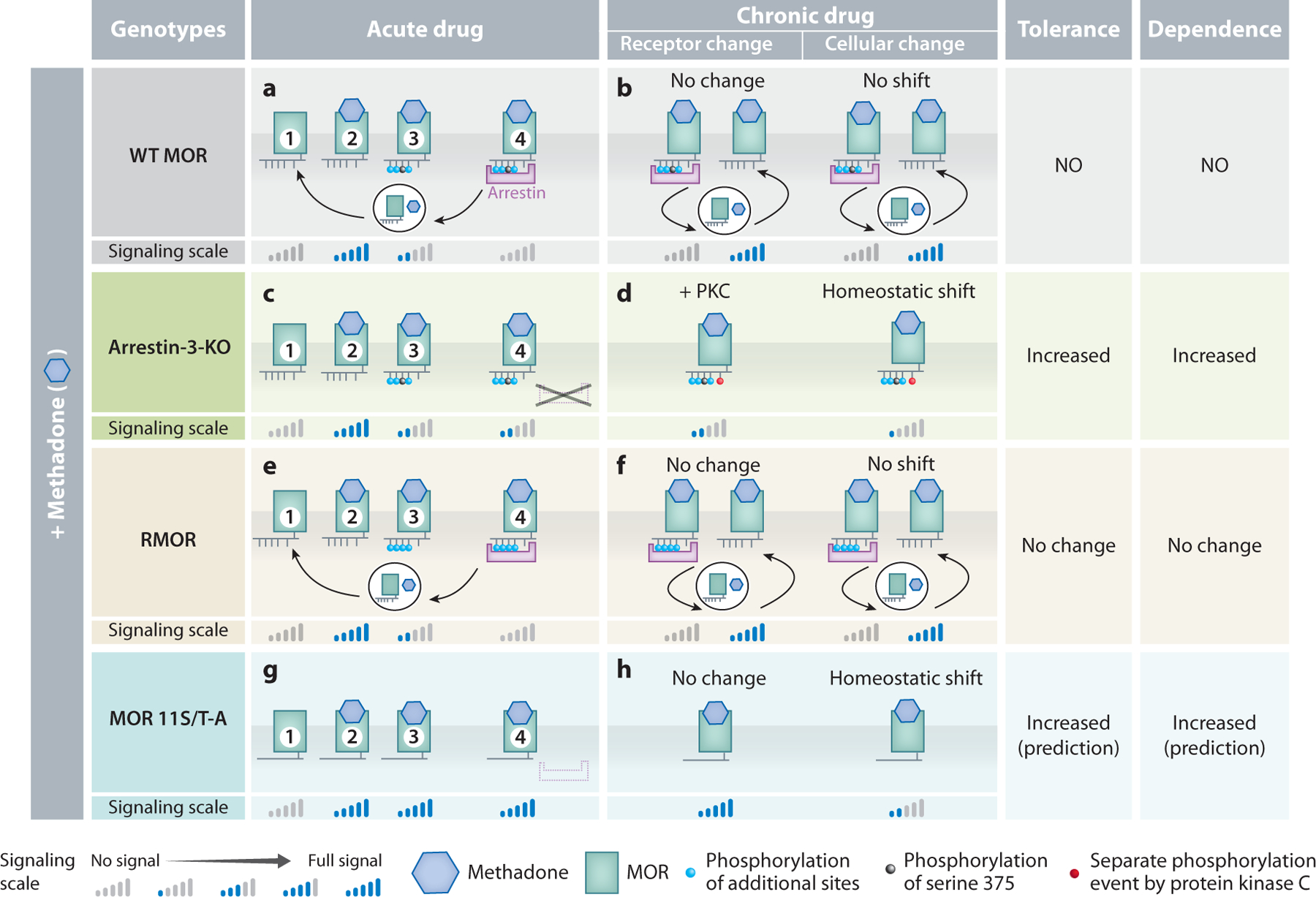
Model of desensitization mechanisms and cellular homeostatic shift in response to acute and chronic methadone in four genotypes of mice: WT, arrestin-3-KO, RMOR knock-in, and MOR 11S/T-A knock-in. (*a*,*b*) Responses to methadone. (*a*) Signaling cycle of the MOR in response to acute methadone: (①) empty, (②) methadone-occupied, (③) phosphorylated by GRK, and (④) arrestin-bound, endocytosed, and recycled. WT MORs are phosphorylated on four residues, desensitizing receptor signal (③). Arrestin-3 recruitment to phosphorylated receptors further desensitizes signal (④). Receptors are (①) endocytosed and recycled, where they (②) bind to ligand and initiate another signaling cascade. (*b*) Chronic methadone. While receptors constantly cycle on and off, signaling remains unchanged with no additional phosphorylation and no homeostatic shift. (*c*) Signaling cycle of the WT MOR in response to acute methadone in arrestin-3-KO mice. (③) WT MORs are phosphorylated on 4 residues, desensitizing receptor signal as in WT mice, but there is no further desensitization by arrestin-3 and no endocytosis. (*d*) Chronic methadone. Without arrestin-3 titration, homeostatic adaptations will occur due to the persistent low signal. This explains why arrestin-3-KO mice develop tolerance to methadone, but WT mice do not. (*e*,*f*) Signaling cycle of the RMOR in response to acute and chronic methadone resembles what occurs with morphine (Figure 7e,f). (*g*,*h*) These are predictions because this experiment has not been reported. (*g*) Signaling cycle of MOR 11S/T-A knock-in mice with acute methadone. MOR 11S/T-A will not be phosphorylated (③) nor recruit arrestin (④). (*h*) Chronic methadone. The persistent signaling will promote homeostatic adaptations, reducing signal and causing increased tolerance and dependence compared to that in WT. Abbreviations: GRK, G protein–coupled receptor kinase; KO, knockout; MOR, mu-opioid receptor; PKC, protein kinase C; RMOR, recycling MOR; WT, wild-type.

Opioid drugs such as morphine cause incomplete phosphorylation of the receptor (62). The poor arrestin-3 recruitment that follows is sufficient to cause partial desensitization of receptors on the membrane, especially with prolonged morphine treatment (105–109), but not to promote endocytosis and rapid resensitization. Balanced compounds such as met-enkephalin and DAMGO actually promote acute desensitization more completely than morphine, but the receptors are then rapidly recycled and resensitized (110). Desensitization of GIRK activation by met-enkephalin occurs normally in arrestin-3 knockout mice (111), suggesting that phosphorylation alone may be sufficient for desensitization in some cases. It seems paradoxical that morphine produces desensitization and tolerance but not internalization, while enkephalin promotes both desensitization and internalization but not tolerance, until we consider the phosphorylation state of the MOR. While the degree of receptor phosphorylation affects the degree of arrestin-3 recruitment, it also likely affects the amount of arrestin-independent (but phosphorylation-dependent) desensitization. As mentioned above, not all opioid ligands promote the same degree of MOR phosphorylation (62). With enkephalin (or methadone), there is phosphorylation of the entire MOR barcode (Figure 8a), which alone desensitizes MOR signaling and promotes arrestin-3 recruitment that shuts signaling down further. The receptor is then endocytosed, recycled, and resensitized to agonist. Signaling at each individual receptor cycles between fully on and fully off but, because this is cyclical, the population always contains fully active receptors (Figure 8a). With morphine, there is phosphorylation of the MOR only on serine 375 (62), which partially desensitizes MOR signaling and produces only weak arrestin-3 recruitment (Figure 7a). Because the receptor is not endocytosed and recycled, signaling of each morphine-bound receptor is suspended in this partially but not fully desensitized state. This could explain why knockout of arrestin-3 increases morphine analgesia but does not alter methadone analgesia (112). Although our understanding of how MORs are dephosphorylated is incomplete, ligands (e.g., DAMGO) that induce rapid phosphorylation and rapid endocytosis cause rapid dephosphorylation (113). In contrast, ligands (e.g., SR-17018) that promote slow kinetics of phosphorylation (113), minimal arrestin recruitment (80), and diminished endocytosis (82, 83) cause prolonged phosphorylation (113) and likely prolonged desensitization.

Repeated morphine treatment likely promotes additional receptor phosphorylation of partially desensitized MORs to further shut them down (105). Rather than GRKs, this is seemingly mediated by protein kinase C (PKC), which has been shown to phosphorylate MORs in response to repeated (not acute) morphine but not DAMGO (114) (Figure 7b,d). Desensitization from chronic morphine treatment is also more persistent than desensitization from an acute dose (100), perhaps reflecting this additional phosphorylation event that is not removed through endocytosis and recycling or changes in the recycling rate through G protein activity (115) and/or cAMP levels (116). There are therefore ample data suggesting that MOR desensitization by both GRKs and arrestins contributes to acute receptor desensitization and some evidence that tolerance to prolonged agonist engages both this and other mechanisms such as PKC. Deleting arrestin-3 would prevent a subset of the desensitization mechanisms and allow more MORs to remain active on the membrane (Figure 7c,d), as would preventing any GRK or PKC phosphorylation (Figure 7g,h). It is therefore not surprising that genotypes lacking MOR c-tail phosphorylation sites or with systemic arrestin-3 deletion show a potentiation of morphine analgesia and some protection from analgesic tolerance to repeated morphine. The partial desensitization produced by morphine, unreversed by endocytosis and resensitization, can also explain the enhanced morphine analgesia in RMOR mice (Figure 7e). Because the RMOR undergoes complete phosphorylation, rapid endocytosis, and resensitization, partially desensitized receptors do not remain on the membrane acting as a sink for available ligand. In the original RMOR paper, this was the mechanism we proposed. In support of this hypothesis we demonstrated that a single morphine dose produced MOR desensitization in the brain stem of wild-type but not RMOR mice (63).

Because tolerance takes days to develop in vivo, rapid receptor desensitization is likely not the sole cause. Tolerance can also result from homeostatic adaptations, even when receptor integrity is unaffected (Figure 5). For example, changes in the availability of second messengers downstream of transducer activation will change the system’s response to a given concentration of agonist. Protracted MOR activation is known to increase levels of cAMP, which opposes MOR-mediated adenylyl cyclase inhibition. Our work provides evidence that rapid desensitization, endocytosis, and recycling of the MOR prevent this homeostatic shift and also circumvent receptor desensitization by allowing for frequent turnover of active receptors (Figures 7e,f and 8a,b). This explains why there is tolerance to morphine (Figure 7a,b) but not methadone (Figure 8a,b) in wild-type mice and tolerance to neither morphine (Figure 7e,f) nor methadone (Figure 8e,f) in RMOR mice. Another group recently showed that morphine sensitivity can be restored by intrathecal DAMGO injection in the rat (117), further suggesting that receptor turnover is antagonistic to tolerance formation. This is similar to how rotation with methadone has previously been used in human medicine to achieve better pain control, although the mechanism was unknown (118). In short, tolerance reduction and increased analgesia have both been observed when arrestin-3 activity is removed and when it is enhanced. We view these seemingly contradictory observations as evidence that both the prevention of receptor desensitization and the enhancement of resensitization can result in similar effects when measured at the level of in vivo drug responses.

Desensitized receptors can contribute to tolerance by lessening the signaling effect of an agonist when it is present. However, receptor desensitization alone cannot account for the opioid withdrawal effect. Although tolerance and analgesia are both determined in the presence of drug, dependence must be measured in the drug’s absence. We define dependence as behavioral effects that are not present in the naïve animal or while the drug is being given at sufficient doses. We refer to this battery of effects as withdrawal, and it is precipitated either by cessation of opioid administration or by giving a competitive antagonist such as naloxone (38) (Figure 4). The onset of withdrawal symptoms when a drug is removed directly implies that these symptoms were being suppressed in the drug’s presence, a task that a silent/desensitized receptor would not accomplish. The appearance of dependence is evidence that mechanisms downstream of the receptor are working in opposition to the opioid signaling cascade (Figure 5j). We propose that adaptations such as cAMP superactivation contribute to tolerance by compensating for signaling that comes from receptors that are still functional and not properly titrated through endocytosis. They also cause dependence by inciting the cell’s hyperactive state that results from agonist removal and subsequent silencing of those functional receptors. For example, we have demonstrated that inhibiting cAMP activity in the VTA during naloxone-precipitated opioid withdrawal prevents withdrawal symptoms, and RMOR mice do not show the cAMP-dependent changes in VTA plasticity in response to morphine that wild-type mice do (65). Critically, RMOR mice show both reduced tolerance and reduced dependence, likely because both partial desensitization and homeostatic adaptations are absent in these mice (Figure 7f). In contrast, only the partial desensitization is prevented in arrestin-3 knockout and MOR-11S/T-A mice (Figure 7d,h), which explains why they still display dependence, in some cases exacerbated compared to wild-type mice. By this model, balanced ligands should show reduced tolerance and dependence (Figure 8b), as we see with morphine in the RMOR mice (Figure 7f), while ultra-G-biased ligands should reduce only tolerance, as we see with morphine in the arrestin-3 knockout and MOR-11S/T-A mice (Figure 7d,h). By extension agonists that are balanced in wild-types (like methadone) and do not produce tolerance and dependence should produce more tolerance and dependence in MOR-11S/T-A and arrestin-3 knockout mice compared to wild-type mice, as the homeostatic shift will be engaged (Figure 8d,h). Given the prominent role that dependence plays in the experiential side of drug abuse and the transition from therapeutic drug taking to the behavioral components of OUD, its clinical relevance should not be ignored. For these reasons, we endorse a research goal that prioritizes a signaling profile that mimics that of endogenous agonists, preventing both tolerance and dependence.

## OPIOID RESEARCH MUST PRIORITIZE HUMAN OUTCOMES

In closing, we are compelled to point out that the search for novel G-biased ligands has transpired with remarkably little attention to how tolerance and abuse liability impede the utility of clinical opioids. This push discounted reports that arrestin-3 knockout mice show enhanced morphine reward and no reduction in morphine dependence in favor of the goal to reduce OIRD. Furthermore, the observed respiratory effects of these new agonists were often conflated with other side effects like tolerance, reward, and dependence, fueling the drive for ultra-G-biased ligands at the expense of any other approach. While none of the common side effects of these drugs are trivial when considering their impact in individual scenarios, we should prioritize these effects based on their relevance in the greater public health context. The respiratory danger posed by opioid drugs is highly relevant once dose escalation and/or the cycle of opioid abuse begins but largely irrelevant when they are given at standard clinical doses under the direct supervision of a physician, as is the case with all novel therapeutics. We know that lethal overdose frequently involves illicitly obtained substances, the possession of which is largely affected by availability and affordability. Therefore, the context in which OIRD is the most influential opioid side effect is unlikely to be directly impacted by the introduction of a new drug to the market, as existing systems of illicit drug access will remain intact. Given our current understanding of how frequently illicit drug use is precipitated by medical use of addictive substances, we are obligated to be vigilant against the manufacture and distribution of new drugs with potentially increased risk for tolerance, dependence, and abuse behavior. An agonist with minimal respiratory effect but high dependence risk is not an improvement over the current options if it ultimately increases the number of people at risk for lethal overdose. Furthermore, OUD still carries deep social repercussions resembling those seen in less acutely lethal substance use disorders. For these reasons, we believe it is critical to center tolerance and dependence prevention in the push for next-generation analgesics.
